# Flatness Prediction of Cold Rolled Strip Based on Deep Neural Network with Improved Activation Function

**DOI:** 10.3390/s22020656

**Published:** 2022-01-15

**Authors:** Jingyi Liu, Shuni Song, Jiayi Wang, Maimutimin Balaiti, Nina Song, Sen Li

**Affiliations:** 1 College of Sciences, Northeastern University, Shenyang 110819, China; wangjiayi@newlandcomputer.com; 2 College of Information Science and Engineering, Northeastern University, Shenyang 110819, China; 2070739@stu.neu.edu.cn (M.B.); 20194447@stu.neu.edu.cn (N.S.); 20194379@stu.neu.edu.cn (S.L.)

**Keywords:** cold rolled strip, deep neural network, Swish activation function, non-convex optimization, batch normalization

## Abstract

With the improvement of industrial requirements for the quality of cold rolled strips, flatness has become one of the most important indicators for measuring the quality of cold rolled strips. In this paper, the strip production data of a 1250 mm tandem cold mill in a steel plant is modeled by an improved deep neural network (the improved DNN) to improve the accuracy of strip shape prediction. Firstly, the type of activation function is analyzed, and the monotonicity of the activation function is deemed independent of the convexity of the loss function in the deep network. Regardless of whether the activation function is monotonic, the loss function is not strictly convex. Secondly, the non-convex optimization of the loss functionextended from the deep linear network to the deep nonlinear network, is discussed, and the critical point of the deep nonlinear network is identified as the global minimum point. Finally, an improved Swish activation function based on batch normalization is proposed, and its performance is evaluated on the MNIST dataset. The experimental results show that the loss of an improved Swish function is lower than that of other activation functions. The prediction accuracy of a deep neural network (DNN) with an improved Swish function is 0.38% more than that of a deep neural network (DNN) with a regular Swish function. For the DNN with the improved Swish function, the mean square error of the prediction for the flatness of cold rolled strip is reduced to 65% of the regular DNN. The accuracy of the improved DNN is up to and higher than the industrial requirements. The shape prediction of the improved DNN will assist and guide the industrial production process, reducing the scrap yield and industrial cost.

## 1. Introduction

Cold rolled strips with high-quality surface, dimension, flatness, and mechanical properties are used in construction, automobiles, household appliances, packaging, building materials, etc., the output and quality level of which reflects the technological strength of a country’s iron and steel industry [1]. With the improvement of industry requirements for the quality of cold rolled strips, the quality of strip shape has become one of the most important indexes to evaluate cold rolled strips. The strip shape refers to the warpage degree of the plate, and mass refers to the distribution of residual stress in the strip. The composition of the strip shape includes the cross-sectional geometry and flatness of the strip in a natural state. Therefore, the quantitative description of plate shape involves multiple indicators in these two aspects, including convexity, wedge, edge thinness, local high points, and flatness [2].

The setting and calculation of strip shape are related to the rolling force and roll bending force required for the rolling deformation of the steel strip. The change of the bending force for the work roll and intermediate roll could deform the roll and change the value of the roll gap; the change of roll gap directly affects the flatness of the strip. Tilt and translation could cause pressure to change the roll, which also affects flatness. Claire et al. [3] proposed an effect function to describe the adjustment effect of the unit adjustment for the adjustment method of the flatness deviation. Wang et al. [4] enriched the effect function in the control system for the flatness of the cold rolling mill and proved that the defined effect function and distribution of flatness deviation were consistent in the form of expression. Chen et al. [5] established the finite element model of a six-high rolling mill based on the effect function to calculate the horizontal and vertical stiffness of the rolling mill. They obtained the influence of the change in values of the no-load roll gap and the bending force of the work roll and intermediate roll on the exit thickness and exit plate crown, which improved the lack of a priori coefficient. However, the effect function could not reflect the flatness in real-time. Therefore, the real-time control model of flatness was proposed by calculating the real-time strip shape during the production process based on the control coefficient of the model. Liang et al. [6] proposed a flatness feed-forward control model which adjusted the corresponding compensation value for the roll bending force by changing the rolling force, regarding it as the pre-control of the closed-loop feedback control system for flatness. The sum of the squares, regarding the difference between the change of the rolling force and the adjustment of the pressure distribution in the loading gap between the work roll bending roll and the intermediate roll bending roll pair, was minimized by minimizing the objective function, thereby controlling the flatness. To make the objective function smaller, Wang et al. [7] proposed a shape control efficiency coefficient based on the feed-forward control model, which refers to a unit change in the shape of the bearing roll gap caused by the effect of the shape actuator. This can be used to calculate the elastic deformation value of the roller system via the influence function method and the difference method. Liu et al. [8] proposed a fuzzy neural network based on a dynamic efficiency matrix to predict control efficiency coefficients. Based on obtaining the control efficiency coefficient by putting the control means into the neural network. This network was proposed to use the rolling force, strip width, actual strip flatness deviation, and actual position change of the actuator as the input, and the efficiency coefficient of 20 control points as the output to train the shallow neural network, further improving the prediction accuracy [9]. However, deep learning (DL) brought new research methods to the study of strip shape. Surface defect detection [10] was also an important research direction, involving a convolutional neural network (CNN) [11,12,13,14,15,16], deep belief network (DBN) [17], visual geometry group network (VGG) [18], and the combination of DL and an extreme learning machine [19,20,21].

The control of strip shape is a complex physical process, and there is a large amount of data in the strip production process. Data in the strip tandem cold rolling process is combined with deep learning technology to establish a predictive model. Through the self-learning of data, many hidden and complex knowledge patterns were obtained, which benefited from investigations regarding the influence of numerical change on the flatness control means of the work roll and intermediate roll, such as the bending force, roll tilt, and roll traverse, on the final exit shape during the tandem cold rolling process. The transformed adjustment of the strip shape during the production process facilitates pre-production control and regulation regarding the input prediction of the strip shape. By changing the input value continuously, more ideal results of flatness prediction are obtained, and the exit shape is controlled effectively. The model could give full weight to the value of data accumulated on the production line, realize data-driven production, and forecast predictions before the start of production. According to the target flatness, each value of the regulation mean is adjusted in advance, reducing the adjustment required during the production process. Therefore, production costs could be saved, and the quality of the flatness and the yield rate of the strip could be improved. In addition, the monotonicity of the activation function is proven unnecessary through the non-convex loss function in the deep learning algorithm. In this paper, the Swish activation function is further improved and optimized, the training and calculation speed of the model is increased, and a higher precision of prediction is realized. Python was used to obtain all of the results and graphs in this paper.

The rest of this paper is organized as follows: Section 2 presents a brief review of the basic concepts and related work of the deep neural network, Section 3 describes the proposed improved deep neural network, Section 4 reports and analyzes the experimental results, and Section 5 summarizes the conclusion.

## 2. Related Theory

### 2.1. Deep Neural Network

A deep neural network(DNN) is an architectural model in deep learning. A feed-forward neural network is composed of an input layer, multiple hidden layers, and an output layer. The layers of the DNN and the neurons between the layers are fully connected; however, the neurons within the layers are not connected. The training of the DNN is divided into forwarding propagation calculation and backpropagation derivation.

Suppose the input samples are $X=\left(X_{1},X_{2},\cdots ,X_{n}\right)$, each sample has *m* features, and the actual output $Y=\left(y_{1},y_{2},\cdots ,y_{n}\right)$ is known. Suppose the output of the *l*-th layer is $a^{l}$ and the input layer is the 0-th layer. This could obtain $a^{0}=X$. Each hidden layer contains two parameters, namely the weight *W* and the bias *b*. The weight *W* is a matrix whose dimension is determined by the output dimension *j* of the previous layer and the number of hidden units *k* in this layer; that is, *W* is a matrix with *j* rows and *k* columns. The *b* is a 1 × *k* vector. The output layer, the same as the hidden layer, contains these two parameters. Each layer will calculate the output of the previous layer. The calculation is divided into two parts. The calculation of the *l*-th layer is:

(1) $$Z^{l}={\left(W^{l}\right)}^{T}a^{l-1}+b^{l}$$

(2) $$a^{l}=\sigma \left(Z^{l}\right)$$

where *W^l^* is the weight of the *l*-th layer, *T* represents the transpose of a matrix, *b^l^* is the bias of the *l*-th layer, *a^l−^*^1^ is the output of the previous layer, and σ is the activation function. The output *a^l^* calculated by the *l*-th layer is regarded as the input of the next layer for calculation until the output layer calculates the predicted value $\hat{y}$. The calculation process of the output layer is the same as that of the hidden layers.

The forward propagation calculation of a sample is completed, and the next step is the backward propagation derivation: the forward propagation calculation obtains the predicted value $\hat{y}$, which has some difference with the sample’s true *y* value; the difference is measured by the loss function *L* which needs to be selected according to the data type and the final purpose, then it can be expressed as $L\left(y,\hat{y}\right)$. The goal of DNN training is to minimize the difference and improve the prediction accuracy of the final model. Therefore, the goal of backpropagation is to update the parameters of the previous layers to continuously reduce the error.

### 2.2. Type of Activation Function

The activation function is an important part of the neural network. The activation function of a hidden layer is generally the same from layer to layer. The main purpose of the activation function is to provide nonlinear modeling ability.

(3) $$a^{l}={\left(W^{l}\right)}^{T}a^{l-1}+b^{l}$$

(4) $$\begin{array}{l} a^{l+1}={\left(W^{l+1}\right)}^{T}a^{l}+b^{l+1}\\ \;\;\;={\left(W^{l+1}\right)}^{T}\left({\left(W^{l}\right)}^{T}a^{l-1}+b^{l}\right)+b^{l+1}\\ \;\;\;=\tilde{W}a^{l-1}+\tilde{b} \end{array}$$

No matter how many layers of the neural network are calculated, if there is no activation function, the final output result is still the same, $\tilde{W}a^{0}+\tilde{b}$. The multi-layer calculation of neural networks is equivalent to that of only one layer. Therefore, DNN has the ability of hierarchical nonlinear mapping with the addition of the activation function [19,20]. The common activation functions are shown in Table 1.

### 2.3. Loss Function

The loss function is used to measure the difference between the predicted value $\hat{y}$ and the true value *y*. On the one hand, the loss function is used in the training process of the model. The loss function is also used as the objective function of the model and through the optimization method, continuously reduces the loss function to update the network parameters to achieve the optimization process of the objective function. On the other hand, it is used to evaluate the prediction results of the final model after training. The smaller the loss value, the better the prediction effect and the higher the accuracy.

During the regression of the DNN, the mean square error loss function is generally used as the objective function to train the model, and MSE is used to evaluate the final model after training. In the classification model of DNN, the cross-entropy loss function is generally used as the objective function to train the model and precision and recall are used to evaluate the classification results. The mean square error (MSE) loss function is used in this study, and its implementation formula is as follows:

(5) $$L\left(y,\hat{y}\right)=\frac{1}{n}\sum_{i=1}^{n}{\left(y_{i}-{\hat{y}}_{i}\right)}^{2}$$

## 3. Improved Deep Neural Network

The activation function is an indispensable core part of the neural network. Regardless of whether the neural network has a DNN, RNN, or convolutional neural network (CNN) structure, the hidden layer and the output layer require an activation function, making the neural network capable of nonlinear modeling. First, it is concluded that the monotonicity of the activation function in the DNN is unnecessary by analyzing the non-convexity of the loss function (Appendix A). When the non-convex loss function can take the minimum value, it will be discussed (Appendix B). Then the advantages of the ELU and Swish function were integrated to improve the Swish function. A performance test was facilitated to investigate the improved activation function on a scientific dataset.

The motivation for improving the activation function comes from the idea of batch normalization. Batch normalization standardizes the output of each layer of the DNN; the mean value of the output is 0, and the variance is 1.

(6) $$u=\frac{1}{n}\sum_{i=1}^{n}z^{\left(i\right)}$$

(7) $$\sigma^{2}=\frac{1}{n}\sum_{i=1}^{n}{\left(z^{\left(i\right)}-u\right)}^{2}$$

(8) $$z_{normalization}^{\left(i\right)}=\frac{z^{\left(i\right)}-u}{\sqrt{\sigma^{2}+\varepsilon }}$$

The advantages of batch normalization are as follows: First, it reduces the deviation of the internal covariates for the output of each layer in the network, making the update of weight more robust, especially in deep neural networks. Therefore, the weight of the back layer for the network is more inclusive of the weight of the front layer. This means that the weight change of the front layer has little effect on the weight of the back layer, and the overall network is more robust; thereby the generalization ability of the network can be improved. Secondly, it normalizes the output of each layer so that the gradient is in an unsaturated region, which can effectively avoid the problems of gradient explosion and vanishing gradient. Finally, the consistency of the overall weight update is maintained, which effectively accelerates the network training process.

Combining the advantages of the ELU activation function, the non-monotonic activation function, Swish, is improved as follows.

(9) $$f\left(x\right)=\{\begin{array}{c} x,\;\;\;x\geq 0\\ \lambda xe^{x},\;x<0 \end{array}$$

The improved Swish activation function is non-monotonic. On the positive half-axis of the *x*-axis, the gradient remains at 1 and greater than the gradient of the Swish function, making the gradient drop faster. On the negative half-axis of the *x*-axis, the absolute value of the negative gradient is increased so that a faster gradient descent speed is maintained on the negative semi-axis, and the output on the negative semi-axis is negative. The parameter λ is used to adjust the magnitude of the negative output, and the average output value is 0, which speeds up training.

A neural network with an 0 output mean value activation function can be called a self-normalized neural network, which realizes the batch normalization through the activation function, making the training process easier. There is no need to extract the output of each layer for normalization to reduce the error rate of the operation and simplify the calculation. In the improved Swish activation function, the method of batch normalization is used for reference, and the specific value of the parameter λ is calculated. By making the mean value for the output of the improved Swish activation function 0, the effect of batch normalization is achieved. Assuming that the input of each layer is a standard normal distribution with a mean value of 0 and a variance of 1, the initial value for the weight of each layer obeys the standard normal distribution, and a normal random number is generated to obtain λ=2.9046. Therefore, the improved Swish activation function formula is as follows.

(10) $$f\left(x\right)=\left\{ \begin{array}{l} x,\;\;\;\;\;\;x\geq 0\\ 2.9046xe^{x},\;\;x<0 \end{array}\right.$$

The improved Swish activation function is derivable when x≠0. The derivative of the function is set to 1 when x=0. The improved Swish activation function draws on the advantages of the ELU and Swish functions and satisfies the conditions as the activation function of the DNN while maintaining a large gradient. It can speed up the training and make the mean value of output 0 to achieve batch normalization. The effect speeds up the training speed while preventing gradient explosion and disappearance, exerting a better effect in the DNN.

## 4. Experimental Results and Discussion

### 4.1. Description of Data

The dataset is the measured data in the actual production of the cold rolling mill, which comes from the five stands in the first production sequence. The established model aimed to predict the final export shape of cold rolled strip steel through various influencing factors. Therefore, the work roll bending force, intermediate roll bending force, rolling force, tension, crimp tension, intermediate roll transverse displacement of the five stands in the first production sequence, and the export flatness measured in each sensor area of the first stand are taken as the input variables, and the export flatness measured in each sensor area of the fifth stand is taken as the output variables.

After deleting the invalid data, empty data, and scrambled data in the dataset, there are 234,527 rows and 86 columns of valid data, namely 234,527 sample points. These sample points are obtained according to time measurement, and a set of data is obtained every 0.08 s. The measured dataset may also continue to change when all known variables are almost unchanged because these influencing factors have a continuous impact on the flatness of the plate. Therefore, time is also an important factor. A column of time should be added to the dataset to reflect the change of sample points over time. Since the first 50,000 data were generated in the unstable rolling process and did not belong to the prediction category of sub-modeling, the first 50,000 data were excluded.

After data cleaning and processing, the dataset is firstly segmented. A total of 184,527 data samples are randomly assigned to the training set and the test set according to the ratio of 7:3. Then, the input data is standardized.

Due to the uniform distribution of data in each column, the corresponding $w_{i}$ obtained by training has a small numerical difference. The gradient descent surface of the loss function along the change of *w* and *b* resembles a round bowl. When the loss function is gradient descent, the objective function can also ensure continuous decline, and no oscillation occurs if the learning rate α is large. The gradient descent step becomes larger, and the loss function decreases rapidly, then the training speed can be accelerated.

### 4.2. Experiments Based on MNIST Dataset

The MNIST dataset is a handwritten digitized database with 60,000 examples in the training library and 10,000 examples in the test library. It is a standard scientific dataset for evaluating the performance of an algorithm by the accuracy of the training set and test set and the decline of the loss function. The MNIST dataset is used to evaluate the performance of the improved Swish activation function and compare it with Sigmoid, ReLU, Swish, and ELU activation functions.

**Figure 1:**
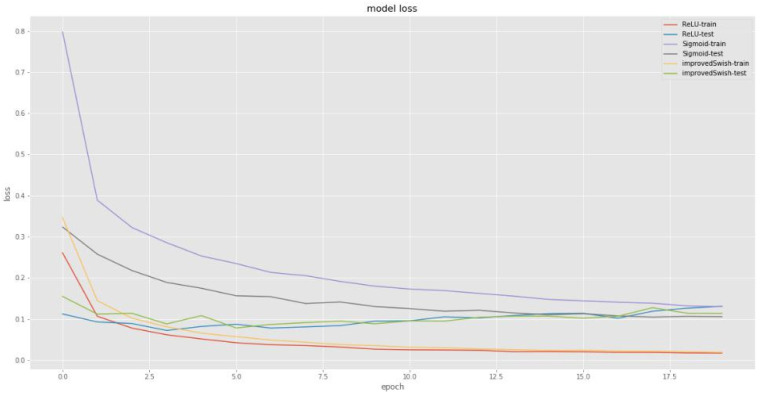
Comparison of loss. The horizontal axis represents epoch times (0~17.5), and the vertical axis represents loss value (0~0.8).

**Figure 2:**
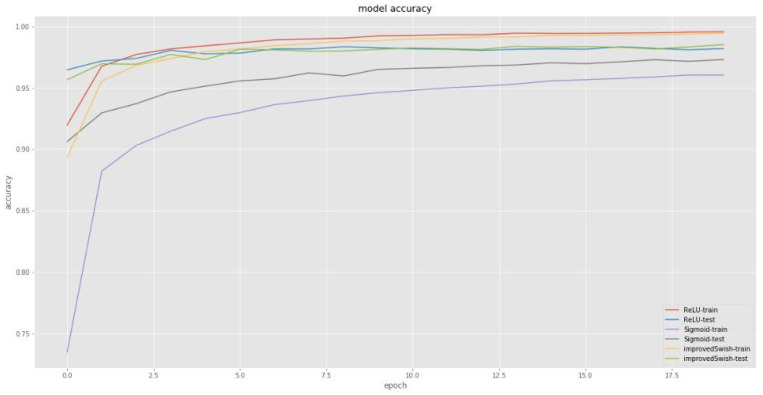
Comparison of accuracy. The horizontal axis represents epoch times (0~17.5), and the vertical axis represents accuracy value (0.7~1).

Figure 1 and Figure 2 compare the loss and accuracy of deep learning models with the improved Swish activation function, Sigmoid function, and ReLU function respectively. The improved Swish and ReLU functions show similar performance in loss reduction and accuracy improvement. However, the improved Swish function’s loss reduction and accuracy improvement are completely superior to the Sigmoid function both in training and test steps.

**Figure 3:**
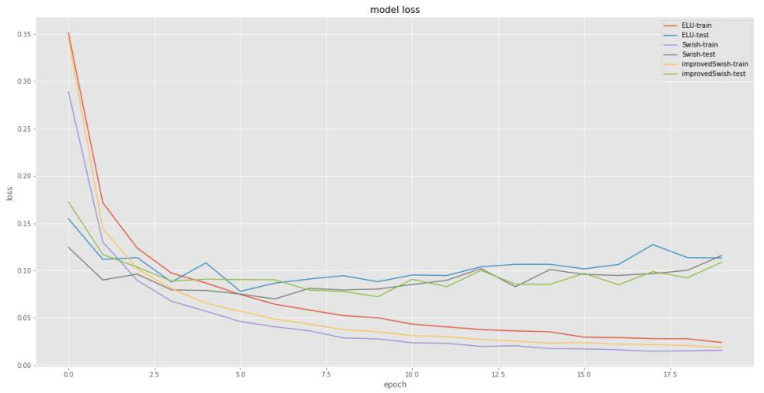
Comparison of loss. The horizontal axis represents epoch times (0~17.5), and the vertical axis represents loss value (0~0.35).

**Figure 4:**
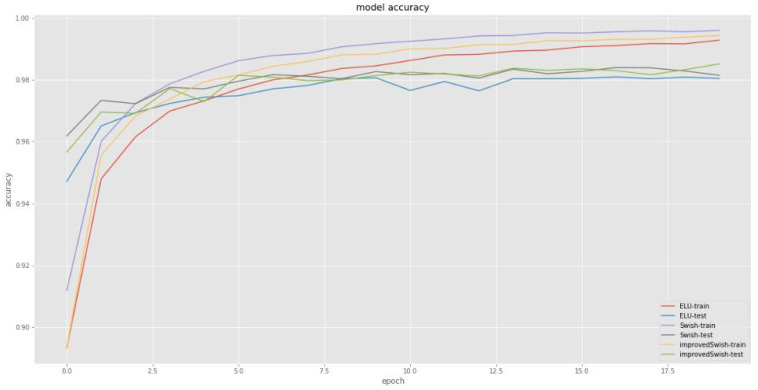
Comparison of accuracy. The horizontal axis represents epoch times (0~17.5), and the vertical axis represents accuracy value (0.8~1).

Figure 3 and Figure 4 compare the loss and accuracy of the deep learning model with the improved Swish, ELU, and original Swish functions, respectively. In terms of model loss, the model with the improved Swish activation function was similar to the training set and test set, compared with the model of the ELU and Swish function; For accuracy, the accuracy of the model with the improved Swish activation function in the training set is slightly lower than that of the model with original Swish function, but higher than that of the model with ELU function, while the accuracy of the model with the improved Swish activation function in the test set is higher than that of the model with the original Swish function and ELU function.

The loss and accuracy of the models with different activation functions on the training set and the test set are shown in Table 2.

The accuracy of the model with the improved Swish activation function on the test set is higher than that of the models with other activation functions. The accuracy on the training set is similar to the original Swish and ReLU function but higher than the ELU and Sigmoid function. The loss of the model using the improved Swish activation function on the test set is smaller than other activation functions. Compared with the current common activation functions, the improved Swish activation function model has achieved good results on the MNIST dataset, and it has better performance in a deep neural network.

### 4.3. Flatness Prediction of Cold Rolled Strip

**Figure 5:**
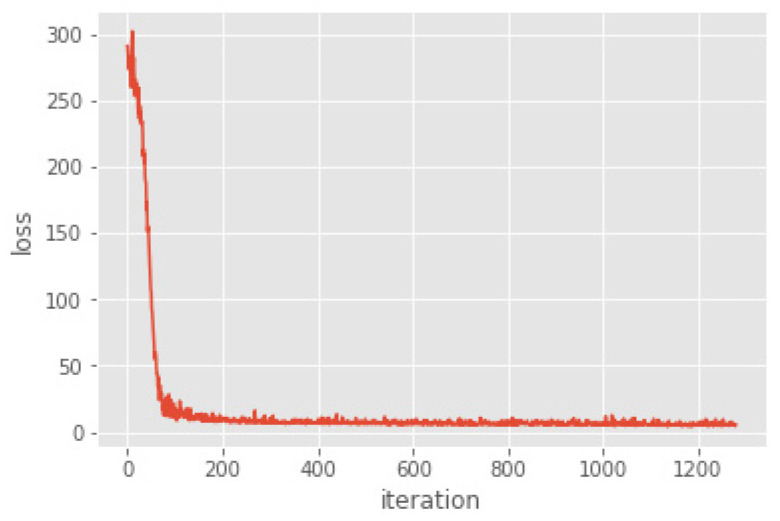
Reduction curve of the loss function.

The DNN with the improved Swish activation function is used to model the cold rolled strip data and analyze the prediction results. The datasets used in the modeling process and the data preprocessing process are detailed in Section 4.1. A total of 20 models are trained, and the model with the smallest MSE in the training set is selected as the optimal model (Figure 5).

In the DNN model with the improved Swish activation function, the MSE loss value of the training set and the test set are 1.281 and 1.305, respectively. The error between them is only 0.024; therefore, the model did not over-fit. Figure 5 show the decline of loss of the model during 1200 iterations. The loss function decreases rapidly in the first 50 iterations, and there is a slight oscillation between 50 and 100 times. After 100 times iteration, the loss value decreased smoothly and then finally converged.

The DNN with the improved Swish activation function was used to predict the flatness of the cold rolled strip, and the predicted value $\hat{y}$ and true value y in each sensor area are compared in Table 3. The difference between the predicted value and the true value of the flatness in the f939 sensor area is large, and the difference in other sensor areas is small. An error of less than 5 % error is the smallest in the f947 sensor area, which is 0.008 %. The MSE of the difference in each sensor area is 1.166, and the prediction accuracy is high.

**Figure 6:**
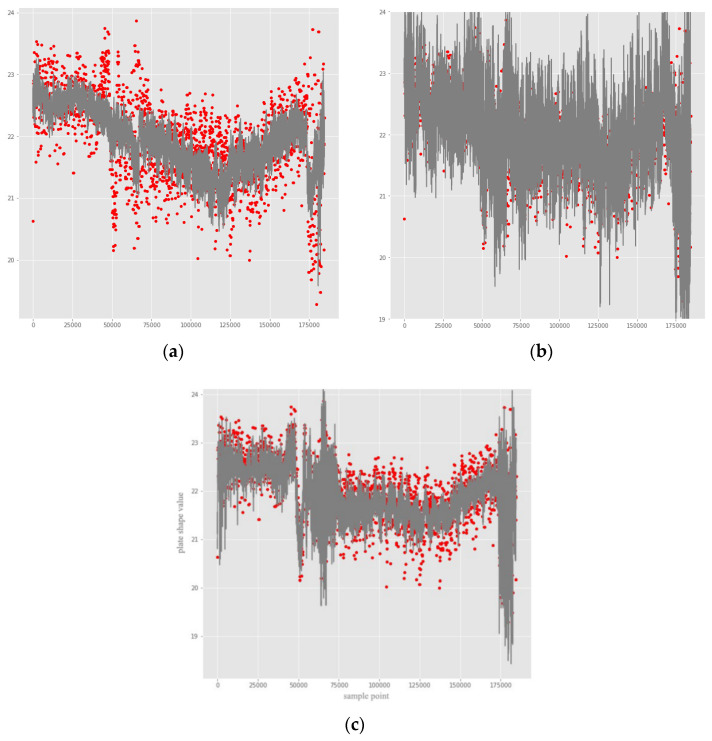
Comparison of real and predicted values. (**a**) BP; (**b**) DNN; (**c**) Improved DNN. The horizontal axis represents sample points (0~175,000), and the vertical axis represents the predicted plastic shape value (18~24).

**Figure 7:**
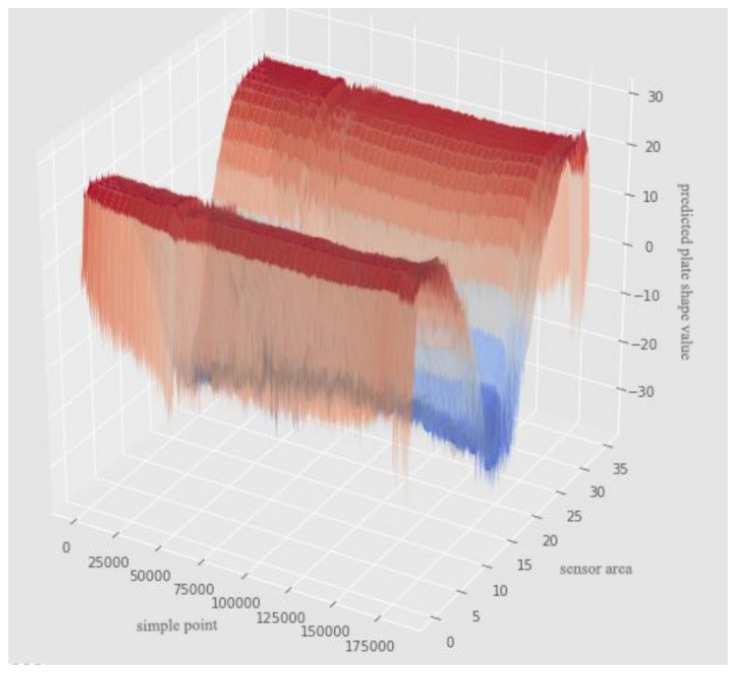
Three-dimensional fitting effect of improved DNN model.

Figure 6 show the comparison between the predicted and true values of the data of the f9314 sensor area. The numerical fitting effect of the DNN with the improved Swish activation function (Improved DNN) in 36 sensor regions is shown in Figure 7. The DNN with the improved Swish activation function has a good fitting ability for the true value, and its predicted value reflects the true value.

The BP, DNN, and DNN with the improved Swish activation function were used to predict the flatness of cold rolled strips (Table 4). MSE of the above models on the training set and test set shows a downward trend, and the DNN model and the DNN with the improved Swish activation function are superior to the BP in terms of prediction ability. Compared with DNN, the DNN with the improved Swish activation function has smaller MSE and higher prediction accuracy. The model can predict the flatness of cold rolled strips accurately and higher than the industrial requirements. The flatness prediction results can assist and guide the industrial production process, reduce the yield of scrap, and reduce the industrial cost.

## 5. Conclusions

In the paper, it is concluded that the non-monotone function can be selected as the activation function by proving the non-convexity of the loss function in the DNN model and the proof that the critical point in the nonlinear deep neural network is the global minimum point was given. Then the parameters of the improved Swish activation function were calculated using the fixed point method and combining the advantages of the ELU and Swish activation functions. The experiments showed that the prediction accuracy of the MNIST dataset is improved by 0.38%. Finally, the DNN with the improved Swish activation function was used to model the actual production data of cold rolled strips. The mean square error for the flatness of cold rolled strip based on the deep neural network with the improved Swish activation function was reduced to 65% of that of the deep neural network. Using this model to predict the export shape of the cold rolled strip can be used to guide the actual production process of cold rolled strip, which is conducive to reducing the scrap rate of the strip and thus reducing the production cost.

## Figures and Tables

**Table 1:** The common activation functions.

Activation Function	Formula	Description
Sigmoid function	$f\left(x\right)=\frac{1}{1+e^{-x}}$	The gradient of the Sigmoid function easily falls into the saturation zone in backpropagation.
Tanh function	$f\left(x\right)=\frac{e^{x}-e^{-x}}{e^{x}+e^{-x}}$	It has soft saturation and vanishing gradient disadvantages.
ReLU function	$f\left(x\right)=\{\begin{array}{c} x\;\;\;\;,\;\;\;\;\;x\geq 0\\ 0\;\;\;\;,\;\;\;\;\;x<0 \end{array}$	As the intensity of the training increases, part of the weight update falls into the hard saturation zone, failing to update.
ELU function	$f\left(x\right)=\{\begin{array}{c} \;\;\;\;\;x\;\;\;\;\;\;\;\;\;\;\;,\;\;\;\;\;x\geq 0\\ \alpha \left(e^{x}-1\right),\;\;\;\;\;x<0 \end{array}$	ELU alleviates the vanishing gradient problem.
Swish function	f(x)=x·sigmoid(βx)	As the amount of data continues to increase, Swish will have better performance.

**Table 2:** Comparison of the best loss and accuracy value for each activation function.

Activation Function	Training Set Loss	Test Set Loss	Training Set Accuracy	Test Set Accuracy
Improved Swish activation function	0.0288	0.1088	0.9928	0.9852
Swish function	0.0279	0.1159	0.9933	0.9815
ReLU function	0.029	0.1307	0.993	0.982
ELU function	0.0308	0.1133	0.9912	0.9802
Sigmoid function	0.1034	0.1066	0.9658	0.9731

**Table 3:** Comparison of real and predicted values.

Sensor Area	Predicted Value	True Value	Sensor Area	Predicted Value	True Value	Sensor Area	Predicted Value	True Value
f939	−3.299	−6.014	f9310	20.291	20.623	9311	23.519	24.176
f9312	23.388	23.659	f9313	23.052	23.336	f9314	22.456	23.036
f9315	21.554	22.622	f9316	19.862	21.321	f9317	17.045	18.237
f9318	13.198	14.230	f9319	8.647	9.440	f9320	3.902	4.500
f9321	−1.152	0.202	f9322	−8.183	−7.844	f9323	−16.903	−19.211
f9324	−23.236	−25.932	f9325	−25.180	−26.736	f9326	−25.405	−25.921
f9327	−26.771	−24.859	f9328	−27.216	−25.265	f9329	−23.888	−24.721
f9330	−16.256	−17.145	f9331	−6.582	−6.235	f940	1.962	2.316
f941	8.507	8.611	f942	13.305	13.130	f943	16.826	16.793
f944	19.659	19.976	f945	21.367	21.737	f946	22.339	22.387
f947	22.915	22.913	f948	23.323	23.340	f949	23.283	23.501
f9410	23.124	23.366	f9411	17.751	17.802	f9412	−7.461	−7.534

**Table 4:** Comparison of the model.

	MSE of Training Sets	MSE of Test Sets
BP	7.851	8.329
DNN	3.229	3.731
Improved DNN	1.281	1.305

## Data Availability

The authors thank the anonymous reviewers for their valuable comments to improve the paper quality.

## Appendix A

### Non-Convexity of the Loss Function

The significance of the monotonic activation function is that a single-layer network can make the loss function with a monotonic activation function a convex function [22]. In the optimization of the convex function, the local minimum would be the global minimum. When using an optimization algorithm such as gradient descent, the local-optimal solution obtained by convergence is the global-optimal solution. After multiple activation functions in the DNN, the loss function could not be strictly convex no matter whether the activation function is monotonous or not. The specific proof is given below:

First, the paper considers a special two-layer DNN. Suppose the activation function is f(z)=z, the weights are $w_{1},w_{2},w_{y}$, then the output is:

(A1) $$f\left(w_{1},w_{2},w_{y}\right)=xw_{1}w_{2}w_{y}$$

Suppose for the loss function $loss\left(f\left(w_{1},w_{2},w_{y}\right)\right)$, the paper obtains the optimal weight $\left(w_{a},w_{b},w_{y}\right)$. The strictly convex function has only one optimum point. If the loss function $loss\left(f\left(w_{1},w_{2},w_{y}\right)\right)$ is a convex function, for any other weight $\left(w_{1},w_{2},w_{y}\right)$,

(A2) $$loss\left(f\left(w_{1},w_{2},w_{y}\right)\right)>loss\left(f\left(w_{a},w_{b},w_{y}\right)\right)$$

Because of

(A3) $$\begin{array}{l} f(w_{1},w_{2},w_{y})=xw_{a}w_{b}w_{y}\\ \;\;\;\;\;=xw_{a}Iw_{b}w_{y}\\ \;\;\;\;\;=xw_{a}AA^{-1}w_{b}w_{y}\\ \;\;\;\;\;=xw_{1}w_{2}w_{y} \end{array}$$

When A and $A^{-1}$ are elementary transformations, there are a series of weights $\left(w_{1},w_{2},w_{y}\right)$ to make $loss\left(f\left(w_{1},w_{2},w_{y}\right)\right)$ take the minimum value. To show whether $\left(w_{1},w_{2},w_{y}\right)$ and $\left(w_{a},w_{b},w_{y}\right)$ are the same point, the following lemma is given.

**Lemma** **A1.**
*For matrix A, A is a zero matrix if there is any full-rank matrix B satisfies AB = A.*

**Proof** **of** **Lemma** **A1.** When A is a 1×1 matrix, that is A,B are constants, B≠0. If *B* is a full-rank matrix, then A=O.

It can be assumed that Lemma 1 holds when A is a k×k matrix. When A is a (k+1)×(k+1) matrix, AB=A can be expressed as a block matrix.

(A4) $$\left(\begin{array}{cc} A^{kk} & A^{k1}\\ A^{1k} & A^{11} \end{array}\right)\left(\begin{array}{cc} B^{kk} & B^{k1}\\ B^{1k} & B^{11} \end{array}\right)=\left(\begin{array}{cc} A^{kk}B^{kk}+A^{k1}B^{1k} & A^{kk}B^{k1}+A^{k1}B^{11}\\ A^{1k}B^{kk}+A^{11}B^{1k} & A^{1k}B^{k1}+A^{11}B^{11} \end{array}\right)=\left(\begin{array}{cc} A^{kk} & A^{k1}\\ A^{1k} & A^{11} \end{array}\right)$$

where A=O is one solution.

For any full-rank matrix B, $B^{k1}=O,B^{1k}=O$, so $B^{11}\neq O$. Formula (A1) can be written as:

(A5) $$\left(\begin{array}{cc} A^{kk}B^{kk} & A^{k1}B^{11}\\ A^{1k}B^{kk} & A^{11}B^{11} \end{array}\right)=\left(\begin{array}{cc} A^{kk} & A^{k1}\\ A^{1k} & A^{11} \end{array}\right)$$

when $A^{kk}=O$ and $B^{11}\neq O$, $A^{k1}=O$, $A^{11}=O$.

Considering $A^{1k}B^{kk}=A^{1k}$, the expression can be:

(A6) $$\left(\begin{array}{c} A^{1k}\\ O^{\left(k-1\right)k} \end{array}\right)B^{kk}=\left(\begin{array}{c} A^{1k}\\ O^{\left(k-1\right)k} \end{array}\right)$$

From the above assumptions, Lemma A1 holds when A is a k×k matrix, then $A^{1k}=O$.

When A is a (k+1)×(k+1) matrix, Lemma A1 holds as well. By mathematical induction, Lemma 1 is proved.

According to Lemma A1, one of the following two situations must be satisfied:

(1) There is a full-rank matrix A such as $w_{a}\neq w_{a}A$, that is $w_{a}\neq w_{1}$. Then $\left(w_{a},w_{b},w_{y}\right)$ is not the only best point, and the loss function is not strictly convex.

(2) Any full-rank matrix A makes $w_{a}=w_{a}A$ or $w_{b}=w_{b}A$, that is $w_{a}$ or $w_{b}$ a zero matrix. No matter what value the remaining parameter $w_{y}$ takes, the output of the network is 0. If it is the optimal value of $\left(w_{1},w_{2},w_{y}\right)$, they are all optimal values for any $w_{y}$. And $\left(w_{1},w_{2},w_{y}\right)$ is not the only optimal value, and the loss function is not strictly convex.

Then the paper considers the ordinary multi-layer neural network. Assuming that the activation function is an arbitrary function α(z) and A is an elementary transformation,

(A7) $$\begin{array}{l} f\left(w_{1},w_{2},w_{y}\right)=\alpha \left(xw_{a}\right)w_{b}w_{y}\\ \;\;\;\;\;\;=\alpha \left(xw_{a}\right)Iw_{b}w_{y}\\ \;\;\;\;\;\;=\alpha \left(xw_{a}\right)AA^{-1}w_{b}w_{y}\\ \;\;\;\;\;\;=\alpha \left(xw_{a}A\right)A^{-1}w_{b}w_{y}\\ \;\;\;\;\;\;=\alpha \left(xw_{1}\right)w_{2}w_{y} \end{array}$$

In the same way, the loss function is not strictly convex.

In summary, the loss function of the multi-layer neural network is not strictly convex regardless of the monotonicity of the activation function. Since the loss function of DNN is non-convex, the activation function does not require monotonicity, and a non-monotonic function can be selected as the activation function. □

## Appendix B

### Global Minimum of the Loss Function

It is difficult to solve the optimal solution for non-convex optimization problems. Although the loss function of DNN is not convex, the critical point is more likely to be a saddle point rather than a false local minimum. Most of the local minimums of a multilayer neural network are at the bottom of the surface for the loss image; it is close to the global minimum. No matter whether it is a local minimum or a global minimum, the result is acceptable when the loss function falls within the range the paper requires. It can be proven that all local minima of the nonlinear deep neural network are global minima under the condition of full rank.

Suppose there are a total of *m* sample points, the input vector is $X\in R^{d_{x}}$, output vector is $Y\in R^{d_{y}}$, the network has *H* hidden layers, the width of each layer is $d_{1},\cdots ,d_{H}$, and $d_{0}=d_{x}$, $d_{H+1}=d_{y}$. Let the weights from the *k*−1 layer to the *k* layer be $W_{k}\in R^{d_{k}\times d_{k-1}},k=1,\cdots ,H+1$. Let $\sigma_{\min}\left(A\right)$ and $\sigma_{\max}\left(A\right)$ be the minimum and maximum singular values of matrix *A* respectively, and the activation function is ψ(x).

(A8) $$L=\frac{1}{m}\sum_{i=1}^{m}\psi \left(W_{H+1}\cdots \psi \left(W_{2}\psi \left(W_{1}X^{\left(i\right)}\right)\right)\right)$$

When a smooth nonlinear function *h* is given, *h* maps the multi-dimensional input to the multi-dimensional output. If each layer of the network is set as a smooth nonlinear function $h_{i}:R^{d_{i-1}}\to R^{d_{i}},\;i=1,\cdots ,H+1$, the entire network can be expressed as a combination of $h_{i}$, that is, *h* can be decomposed into a series of smooth nonlinear functions, namely

(A9) $$h_{\left(H+1\right):1}=h_{H+1}h_{H}\cdots h_{1}$$

Therefore, the goal of deep neural network learning is to find a set of nonlinear functions $h_{1},h_{2},\cdots ,h_{H+1}$ to minimize the overall loss function.

(A10) $$L\left(h\right)=L\left(h_{1},h_{2},\cdots ,h_{H+1}\right)=\frac{1}{2}E\left[{\left\|h_{H+1}h_{H}\cdots h_{1}\left(X\right)-Y\right\|}_{2}^{2}\right]$$

The minimization of the square loss is the conditional expectation of *Y* given *X*, that is $h^{*}\left(x\right)=E\left[Y|X=x\right]$, which divides the loss function into two parts.

(A11) $$L\left(h\right)=\frac{1}{2}E\left[{\left\|h_{H+1}\cdots h_{1}\left(X\right)-h^{*}\left(X\right)\right\|}_{2}^{2}\right]+C$$

where *C* is a constant term, representing the deviation independent of $h_{1},h_{2},\cdots ,h_{H+1}$. If $h_{H+1}h_{H}\cdots h_{1}=h^{*}$, the optimal solution $L^{*}=C$ is obtained at this time.

The following can prove that Theorem 1 holds when the following conditions are met.

(1) Suppose the function space satisfies $F=\left\{h:R^{d_{x}}\to R^{d_{y}}|h(0)=0,\;\underset{x}{\sup}\frac{{\left\|h\left(x\right)\right\|}_{2}}{{\left\|x\right\|}_{2}}<\infty \right\}$, where h∈F can be derived, $F_{i}=\left\{h_{i}:R^{d_{i-1}}\to R^{d_{i}}|h_{i}(0)=0,\underset{x}{\;\sup}\frac{{\left\|h_{i}\left(x\right)\right\|}_{2}}{{\left\|x\right\|}_{2}}<\infty \right\},\;i=1,\cdots ,H+1$, where $h_{1}\in F_{1},\cdots ,h_{H+1}\in F_{H+1}$ can be derived and $h_{H+1}\cdots h_{1}\in F$.

(2) Suppose $d_{x}\geq d_{y},\;d_{i}\geq d_{x},\;d_{i}\geq d_{y},\;i=1,2,\cdots ,H+1$.

**Theorem** **A1.** *If* $h_{H+1:2}\left(Z\right)$*is secondarily differential and* ε>0*such that* $\sigma_{\min}\left(J\left[h_{H+1:2}\right]\left(Z\right)\right)\geq \varepsilon$*holds for any* $Z\in R^{d_{1}}$, *the critical point of* L(h)*is also the global minimum.*

**Proof** **of** **Theorem** **A1.** For $\forall \eta \in F_{i}$, by the definition of Frechet derivative,

(A12) $$\langle D_{h_{i}}\left[L\left(h\right)\right],\eta \rangle =\underset{\varepsilon \to 0}{\lim}\frac{L\left(h_{1},\cdots ,h_{i}+\varepsilon \eta ,\cdots ,h_{H+1}\right)-L\left(h\right)}{\varepsilon }$$

where $D_{h_{i}}\left[L\left(h\right)\right]$ represents the Frechet derivative of the overall loss $h_{i}$, and $\langle D_{h_{i}}\left[L\left(h\right)\right],\eta \rangle$ represents the directional derivative along the direction η. Let J[f](x) denote the Jacobian matrix of function *f* at point *x*, then

(A13) $$\begin{array}{l} \;L\left(h_{1},\cdots ,h_{i}+\varepsilon \eta ,\cdots ,h_{H+1}\right)\\ =\frac{1}{2}E\left[{\left\|h_{H+1:i+1}\left(h_{i}+\varepsilon \eta \right)h_{i-1:1}\left(X\right)-h^{*}\left(X\right)\right\|}_{2}^{2}\right]+C\\ =\frac{1}{2}E\left[{\left\|h_{H+1:i+1}\left(h_{i:1}\left(X\right)+\varepsilon \eta \left(h_{i-1:1}\left(X\right)\right)\right)-h^{*}\left(X\right)\right\|}_{2}^{2}\right]+C\\ =\frac{1}{2}E\left[{\left\|h_{H+1:1}\left(X\right)+\varepsilon J\left[h_{H+1:i+1}\right]\left(h_{i:1}\left(X\right)\right)\eta \left(h_{i-1:1}\left(X\right)\right)+O\left(\varepsilon^{2}\right)-h^{*}\left(X\right)\right\|}_{2}^{2}\right]+C\\ =L\left(h\right)+\varepsilon E\left[{\left(h_{H+1:1}\left(X\right)-h^{*}\left(X\right)\right)}^{T}J\left[h_{H+1:i+1}\right]\left(h_{i:1}\left(X\right)\right)\eta \left(h_{i-1:1}\left(X\right)\right)\right]+O\left(\varepsilon^{2}\right) \end{array}$$

Then

(A14) $$\langle D_{h_{i}}\left[L\left(h\right)\right],\eta \rangle =E\left[{\left(h_{H+1:1}\left(X\right)-h^{*}\left(X\right)\right)}^{T}J\left[h_{H+1:i+1}\right]\left(h_{i:1}\left(X\right)\right)\eta \left(h_{i-1:1}\left(X\right)\right)\right]$$

In the Formula (A1), the paper takes i=1 to obtain

(A15) $$\langle D_{h_{1}}\left[L\left(h\right)\right],\eta \rangle =E\left[{\left(h_{H+1:1}\left(X\right)-h^{*}\left(X\right)\right)}^{T}J\left[h_{H+1:2}\right]\left(h_{1}\left(X\right)\right)\eta \left(X\right)\right]\forall \eta \in F_{1}$$

Suppose $A\left(X\right)=J\left[h_{H+1:2}\right]\left(h_{1}\left(X\right)\right)$, and A(X) is a matrix with row full rank from the hypothesis (2). Then $A\left(X\right)A{\left(X\right)}^{T}$ is invertible. Define a special direction

(A16) $$\tilde{\eta }\left(X\right)=A{\left(X\right)}^{T}{\left(A\left(X\right)A{\left(X\right)}^{T}\right)}^{-1}\left(h_{H+1:1}\left(X\right)-h^{*}\left(X\right)\right)$$

Then

(A17) $$\langle D_{h_{1}}\left[L\left(h\right)\right],\tilde{\eta }\rangle =E\left[{\left\|h_{H+1:1}\left(X\right)-h^{*}\left(X\right)\right\|}_{2}^{2}\right]$$

Prove $\tilde{\eta }\left(X\right)\in F_{1}$ below. If $h_{H+1:1}\left(0\right)-h^{*}\left(0\right)=0$, $\tilde{\eta }\left(0\right)=0$. If $J\left[h_{H+1:2}\right]$ is derivable and $h_{1}\in F_{1}$, $A\left(X\right),\;A{\left(X\right)}^{T},\;{\left(A\left(X\right)A{\left(X\right)}^{T}\right)}^{-1}$ is derivable. If $h_{H+1:1}-h^{*}\in F$, $\tilde{\eta }\left(X\right)$ is derivable. Suppose Singular Value Decomposition (SVD) of A(X) is decomposed into $A\left(X\right)=U\sum V^{T}$, where $\sum =\left[\begin{array}{cc} \sum_{1} & O \end{array}\right]$. Then

(A18) $$\begin{array}{l} A{\left(X\right)}^{T}{\left(A\left(X\right)A{\left(X\right)}^{T}\right)}^{-1}=V\sum^{T}U^{T}{\left(U\sum V^{T}V\sum^{T}U^{T}\right)}^{-1}\\ \;\;\;\;\;\;\;\;\;\;\;=V\sum^{T}U^{T}{\left(U\sum_{1}^{2}U^{T}\right)}^{-1}\\ \;\;\;\;\;\;\;\;\;\;\;=V\sum^{T}U^{T}U\sum_{1}^{-2}U^{T}=V\left[\begin{array}{c} \sum_{1}^{-1}\\ O \end{array}\right]U^{T} \end{array}$$

Suppose the operator norm of matrix *A* is ${\left\|A\right\|}_{OP}=\underset{x}{\sup}\frac{{\left\|Ax\right\|}_{2}}{{\left\|x\right\|}_{2}}$, then

(A19) $${\left\|A{\left(X\right)}^{T}{\left(A\left(X\right)A{\left(X\right)}^{T}\right)}^{-1}\right\|}_{OP}=\sigma_{\max}\left(A{\left(X\right)}^{T}{\left(A\left(X\right)A{\left(X\right)}^{T}\right)}^{-1}\right)\leq \frac{1}{\varepsilon }$$

For ∀h∈F, the paper defines the norm of the nonlinear function *h* as ${\left\|h\right\|}_{nl}=\underset{x}{\sup}\frac{{\left\|h\left(x\right)\right\|}_{2}}{{\left\|x\right\|}_{2}}$,

(A20) $$\begin{array}{l} {\left\|\tilde{\eta }\left(X\right)\right\|}_{2}={\left\|A{\left(X\right)}^{T}{\left(A\left(X\right)A{\left(X\right)}^{T}\right)}^{-1}\left(h_{H+1:1}\left(X\right)-h^{*}\left(X\right)\right)\right\|}_{2}\\ \;\;\;\;\leq {\left\|A{\left(X\right)}^{T}{\left(A\left(X\right)A{\left(X\right)}^{T}\right)}^{-1}\right\|}_{OP}{\left\|h_{H+1:1}\left(X\right)-h^{*}\left(X\right)\right\|}_{2}\\ \;\;\;\;\leq {\left\|A{\left(X\right)}^{T}{\left(A\left(X\right)A{\left(X\right)}^{T}\right)}^{-1}\right\|}_{OP}{\left\|h_{H+1:1}-h^{*}\right\|}_{nl}{\left\|X\right\|}_{2} \end{array}$$

Then

(A21) $${\left\|\tilde{\eta }\right\|}_{nl}\leq {\left\|A{\left(X\right)}^{T}{\left(A\left(X\right)A{\left(X\right)}^{T}\right)}^{-1}\right\|}_{OP}{\left\|h_{H+1:1}-h^{*}\right\|}_{nl}\leq \frac{{\left\|h_{H+1:1}-h^{*}\right\|}_{nl}}{\varepsilon }$$

Then $\tilde{\eta }\in F_{1}$. Because of:

(A22) $$\begin{array}{l} {\left\|D_{h_{1}}\left[L\left(h\right)\right]\right\|}_{OP}\geq \frac{\langle D_{h_{1}}\left[L\left(h\right)\right],\tilde{\eta }\rangle }{{\left\|\tilde{\eta }\right\|}_{nl}}\\ \;\;\;\;\;\;\geq \frac{\varepsilon E\left[{\left\|h_{H+1:1}\left(X\right)-h^{*}\left(X\right)\right\|}_{2}^{2}\right]}{{\left\|h_{H+1:1}-h^{*}\right\|}_{nl}}\\ \;\;\;\;\;\;=\frac{\varepsilon \left(\left|L\left(h\right)-L^{*}\right|\right)}{{\left\|h_{H+1:1}-h^{*}\right\|}_{nl}} \end{array}$$

then

(A23) $${\left\|D_{h_{1}}\left[L\left(h\right)\right]\right\|}_{OP}{\left\|h_{H+1:1}-h^{*}\right\|}_{nl}\geq \varepsilon \left(\left|L\left(h\right)-L^{*}\right|\right)$$

And the critical point of L(h) satisfies ${\left\|D_{h_{1}}\left[L\left(h\right)\right]\right\|}_{OP}=0$, and $L\left(h\right)=L^{*}$. In summary, the critical point that satisfies the assumptions (1) and (2) and Theorem 1 is the global minimum of the loss function, and Theorem 1 is proved. □
